# Personality disorders in medical psychology: medical training and professional creativity: Transtornos de personalidade em psicologia médica: formação médica e criatividade profissional

**DOI:** 10.1590/1516-3180.2014.8651004

**Published:** 2015-12-08

**Authors:** Décio Gilberto Natrielli, Décio Gilberto Natrielli, Anderson Souza Martins da Silva, Michelle Álefe Alves Cury, Vinícius Toledo Couto, Raitza Araújo dos Santos Lima, Bárbara Sousa Modesto

**Affiliations:** I MD. Professor of Psychiatry, Universidade de Santo Amaro (UNISA) and Universidade Paulista (UNIP), and Attending Psychiatrist, Irmãs Hospitaleiras do Sagrado Coração de Jesus, São Paulo, Brazil.; II MD. Scientific Coordinator, Multidisciplinary Medical Psychology Committee, Associação Paulista de Medicina (APM), São Paulo, Brazil.; III MD . Intern, Department of Psychiatry, UNISA, São Paulo, Brazil.

According to the fifth edition of the Diagnostic and Statistical Manual of Mental Disorders^1^(DSM-5), "a personality disorder is an enduring pattern of inner experience and behavior that deviates markedly from the expectations of the individual's culture, is pervasive and inflexible, has an onset in adolescence or early adulthood, is stable over time, and leads to distress or impairment". In this new edition, the American Psychiatry Association has added an alternative model of personality disorder (PD) evaluation that reflects a need for a complimentary view of this psychiatric disorder and addresses "numerous shortcomings" of the current conceptual clinical basis. 

Indeed, these shortcomings appear in all medical fields. Despite technology, evolving knowledge and guidelines, there will always be gaps in human models, whether they are empirical, statistical or subjective paradigms. Thus, PDs are subjective and difficult to measure, and the heuristic values of these disorders are ambiguous because they have a historical background belonging to human development. There is some overlapping among all the theories, but they are difficult to apply in clinical practice. 

In this letter, we give our support to the idea that PDs are among the disorders that incite disagreement within the research and clinical fields, in comparison with other diseases or psychiatric illnesses. From this scenario, one question emerges: How would we measure someone's intimate perception of inner or outer realities? Philosophers are working on this issue, but we psychiatrists are trying to "read thoughts and minds," so as to allocate and classify them into categories. This challenge may be reflected in the practices of clinicians, surgeons, gynecologists, obstetricians, pediatricians, oncologists and so on, who will face patients with all types of behaviors, from eccentric to numb, elated to depressed, impulsive to protracted and anxious to fearless, and other antithetical pairs of adjectives relating to human emotions.

Patients with PDs can be found in different kinds of medical specialties, hospital wards and outpatient settings. Although psychiatry has become a prevalent issue in academic and clinical practice, the nosological category "PD" is still a challenge even to psychiatrists, and is associated with poor treatment outcomes, legal issues, self-injuries, aggressiveness, hostility, staff distress, impairment of the doctor-patient relationship and other variables that may contribute to failure in several realms.

In clinical practice, it is commonly observed that doctors (other than psychiatrists) are not trained to identify patients at risk of or presenting personality traits that would be diagnosed as a PD. For example, pediatricians or clinicians might be faced with evaluating and treating cases like that of an adolescent hospitalized due to an infectious disease who evolves with externalizing behaviors (and whose parents may also have a psychopathological condition). Behavioral disorders of this nature will require an approach that goes beyond physical examination and, instead, evaluates the disturbed adolescent's environmental, familial, genetic and temperamental background. Another example might be a 17-year-old woman in her 36^th^ week of pregnancy who presents suicidal ideation, impulsiveness, mood swings and hostility, previous suicidal attempts, drug use and a current unstable relationship with her boyfriend. These two examples show the skills and patience that pediatricians, clinicians and obstetricians must summon up in order to lead the respective cases towards successful treatment. Referral of the patient and family to a psychiatrist is one of the main procedures that must be followed in order to prevent fatalities.

However, the doctor-patient relationship goes beyond referral to another colleague. Evidence-based medicine provides knowledge about the conceptualization, epidemiology, clinical course and treatment of diseases and mental disorders. In the case of PDs, both the conceptual and the treatment domains are subjective and, indeed, depend on the doctor's ability to come closer to the patient and family and establish a higher level of communication. This higher level is categorized as creativity and relates to symbolic and abstract thought that is characteristic of developed intelligence and cerebral cortical integrity. Its use would strength the patient's confidence in his doctor and solidify the bridge between the two individuals, thereby avoiding impulsiveness and disruptive behaviors.

The article "Relationship between mental health and spiritual wellbeing among hemodialysis patients: a correlation study" by Martínez and Custódio^2^ is a noble attempt to identify and study coping mechanisms among patients in a complex clinical situation that imposes a huge stress load that may interfere in quality of life and mental health. The focus on spirituality is important because in some dimensions of personality traits, like Cloninger's self-transcendence character dimension,^3^ ^4^ the sense and perception of being part of a spiritual or greater universal force could be an adaptive quality of a developed personality, especially when facing suffering, diseases and even death, the inevitable processes of life and aging. Martínez and Custódio's introduction emphasizes this issue: "*Psychiatric disorders are common among hemodialysis (HD) patients and are associated with increased morbidity and mortality, and reduced quality of life. Spirituality is an important factor in the quality of life of HD patients. According to Koenig et al., spirituality is a personal quest to understand aspects of life, its meaning and the relationship with the sacred, which may or may not involve religious practices or formation of religious groups. Spirituality is a potential resource in relation to mental health and is a coping mechanism for stressful experiences *". In the discussion, these authors provide a priceless contribution towards the importance of the doctor-patient relationship, through stating "*The higher the scores for spiritual wellbeing and, especially, for existential well-being are, the higher the likelihood of better mental health is. There are different ways of coping with disease and treatment. Suffering is a personal experience, but it is still possible to extract lessons from suffering and to rethink values, thereby giving life a new meaning *".

It is valid to add that personality encompasses the whole spiritual experience, as defined above, and that the authors Martínez and Custódio^2^ made efforts to "measure" one of the most important domains of human mental experience. With regard to PD patients, they may present clinically with poor spiritual concepts and thus may fail to build resources that would allow them to sustain self-stability during such stressful situations.

What would be the attributes of a medical doctor that would enable an ideal approach towards PD patients? First, we must remember that although an ideal or perfect model belongs to human beliefs and thoughts, it will never be reached or given concrete existence in its entirety. Second, culturally developed humans are always pursuing the perfect model or paradigm. And third, motivation is singular to every individual and its background is hidden behind one's personality, environment and developmental life. Therefore, the attributes include technical evidence-based knowledge and personality skills (including creativity). Collision or bonding of the doctor and patient's personalities may result in infinite reactions, although limited to this bi-personal environment. Perhaps the answer to this question is that there are no guidelines or recipes that doctors can follow, although some medical catchphrases may help, like "in-depth knowledge" and "do no harm". Indeed, as stated implicitly in the concept of medicine, all cases should be considered to be unique and singular, and a health professional's training includes his or her cultural, educational, familial and personal backgrounds.

The relationship between doctor and patient is based on trust, respect and understanding of suffering, while paying attention to the clinical history and detailed anamnesis comprising intrinsic specialty orientation and guidance. Nevertheless, doctors should never underestimate human beings' wishes and their particular ways of dealing with suffering.

Through evidence-based medicine, it is clear that efficient medical care is very dependent on analysis and amalgamation of appropriate clinical data, and on decision-making with regard to the risks and benefits of a diagnostic test or treatment, among other procedures. However, there will be no relationship between the two human beings, in this case a medical doctor and a patient, unless the doctor has the ability to interact in an empathic manner, through taking into account the peculiarities of physical and psychological functioning, listening in a qualified manner, appreciating the complaint, using easily-understood language that helps the patients towards more adaptive behaviors and, finally, identifying the patients' needs and transforming them into "objects of health". In the case of patients with PDs, the professional who conducts the case should be flexible, patient and creative so as to establish a stable bond during the period of care and, at the end of the treatment, provide secure maintenance of this stability through a network of supportive and (multi-)professional care.

To mention "creativity" in a scientific paper might be considered as showing a lack of objectivity and empiricism, thereby impoverishing the value of such studies to the field of medicine. Moreover, if a doctor or researcher mentions his or her "practical experience" without numbers and a statistical basis, the information may not be considered trustworthy. Nonetheless, this sometimes "doubtful" or "unreliable" professional's inner experience, allied with protocols and guidelines, might provide better care for PD patients with severe psychiatric symptoms. Such symptoms could include an unstable sense of self, hopelessness, suicidal ideation, rage, impulsiveness, social withdrawal, compulsive self-destructive behavior, feelings of insecurity, loneliness, desperateness and suspiciousness, among others. Creativity is necessary in order to deal with the anguish and impotence that the professional will face, through the countertransference experienced while attending these enigmatic patients.

Just to make things a little more complicated, some PD patients are prone to view neutral situations as threatening, and may require additional reassurance and assistance in understanding social cues.^5^ This is part of the patient's social cognition and it will not be changed in the blink of an eye. In general, individuals with PD are likely to seek medical assistance and will arrive not only in the psychiatry department but also and more frequently in other specialties. Nonetheless, initial referral to a psychiatrist may be helpful and spontaneous. However, our intention in this letter was to convey the importance of the professional's creativity when applying scientific knowledge. As mentioned before, it is far from inappropriate to describe the quality required as "patience," since these PD patients are really tough, tricky and puzzling. Sometimes the entire healthcare staff may perceive such patients as ungrateful, despite all the efforts that are made to help and alleviate their main sources of suffering. Hence, creativity should be stimulated during medical training, thereby endowing the professional with skills to work around these situations and provide the best care for these patients, while avoiding staff distress, thus improving the treatment outcome.
